# Progress in Cancer

**Published:** 1904-04-09

**Authors:** 


### PROGRESS IN CANCER.

(*Continued from page* 10.)

**A Cause of Malignancy in New Growth.**—Professor Farmer,[6 7 8 9] in a paper read before the Royal Society, communicated the joint observations made by himself, Mr. Moore and Mr. Walker on the subject of malignant growths. These researches go to show that the cell proliferation of cancerous neoplasms is characterised by a nuclear division which is distinct and distinguishable from that which is common to cell proliferation in ordinary tissue growth or in benign tumours. The particular form which is found according to these researches in all malignant growths so far examined, resembles in its essential cytology that which is met with in reproductive tissue. Without entering into details it may be stated generally that the proliferating cells which are typical of malignant growths pass through a stage of nuclear division which is technically known as heterotype mitosis. This heterotype mitosis, which also occurs as a phase in the history of the development in sexual cells, shows prophases of development in which the chromosomes, instead of appearing as delicate thin rods or V's, split longitudinally to present the appearance of short thickened loops or rings. Further, these chromatic loops or rings are arranged lengthwise along the spindle and ultimately divide transversely instead of longitudinally as is normally the case in nuclear division. When once this heterotype mitosis has supervened in the progress of cell proliferation, all the daughter cells arising from such heterotype division contain a reduced number of chromosomes. The type of nuclear division which these observers have found in malignant growths may be said to be the same as is found in the developmental stages of ovaries and testes, with the difference, however, that the series of metamorphic phases stops short of the phase at which true sexual cells—ova and spermatozoa—are produced. A point of interest which attaches to these observations is that their essential features were not discovered by accident. They were anticipated and expected from the analogy of vegetable growths, and the characteristic appearances of the reproduction in ferns. The recognition of heterotype mitosis in new growths, apart from the enormous advantage it confers in the differential diagnosis between malignant and benign neoplasms, also indicates a direction in which future researches with regard to the pathogenesis of cancerous growths may be pursued.

**The Significance of the Zoological Distribution, the Nature of the Mitoses, and the Transmissibility of Cancer.**[10 11]—This work is reported by Bashford and Murray from the laboratories of the Cancer Research Fund. Malignant growths were examined from many different animals, and from many parts of the world, and it was proved that in their clinical, pathological, anatomical and microscopic characters these growths were in all essential features identical with those found in man. The wide diversity in the habitat, food, and mode of life of the animals suggests that such external conditions play only a secondary part in determining the occurrence of malignant growths, and appear to indicate that the cause of cancer must be sought in a disturbance of reproduction and cell life common to all forms of animal life in which it occurs. Hence purely cytological investigations must evidently have a most important bearing on the solution of problems connected with the unrestricted growth of cancer. The same heterotype mitoses were found in the examination of the growths as observed in the previous paper, by Farmer, Moore, and Walker. When an attempt was made to transfer a malignant tumour from one animal to another of the same species it was found that the new tumour which arises from the implantation of a portion of a tumour from another animal, springs from the actual cells introduced, and not from the part into which the portion of tumour was implanted. Drs. Bashford and Murray conclude from their researches that "cancer is an irregular and localised manifestation of a process otherwise natural to the life-cycle of all organisms."

[6] Brit. Med. Jour., Dec. 26, 1904. [7] Lancet, Dec. 26, 1904. [8] Ibid., Jan. 9, 1904. [9] Ibid., Jan. 30, 1904. [10] Brit. Med. Jour., Jan. 30, 1904. [11] Lancet, Jan. 30, 1904.

(*To be concluded.*)

